# Supplementary material for: On the Track of New Endoscopic Alternatives for the Treatment of Selected Gastric GISTs—A Pilot Study
Source: Medicina (Kaunas). 2021 Jun 16;57(6):625. doi: 10.3390/medicina57060625 (PMC8234534; doi:10.3390/medicina57060625)
Supplement: Supplementary file 1 [file medicina-57-00625-s001.zip › medicina-1216653-supplementary.pdf]

Supplementary File 1

STROBE Statement—checklist of items that should be included in reports of observational studies

	Item No	Recommendation	Subheading of article
**Title and abstract**	1	(*a*) Indicate the study's design with a commonly used term in the title or the abstract<br>*The design, case-control study was mentioned in the title and the abstract*	*Title and Abstract*<br>1
		(*b*) Provide in the abstract an informative and balanced summary of what was done and what was found<br>*A structured abstract that includes background, method, results and conclusion parts which contain balanced and informative summary*	*Abstract*<br>1
**Introduction**			
Background/rationale	2	Explain the scientific background and rationale for the investigation being reported<br>*We explained in the background that recently, the number of studies reporting this virus to be neurovirulent has increased, associating it with neurological complications in patients with DF. The most prevalent neurological disorder occurring during DF is encephalopathy. Case study reports found that adult patients with DF exhibit delusions with auditory and visual hallucinations, agitation and psychotic symptoms and fears, agitation, irritable affect, psychosis, mania and catatonia. Among the identified encephalopathies, depressive and anxiety symptoms were the most studied among adult patients with DF. Yet, information among pediatric patients with DF remain inadequate. There is still a pressing need to conduct more studies to measure the impact of DF infection on mental health of pediatric patients.*	*Introduction*<br>1-2
Objectives	3	State specific objectives, including any prespecified hypotheses<br>*This study aimed to estimate the prevalence of depressive and anxiety symptoms among pediatric in-patients with DF and compare it with that among healthy school-based youth controls. We also sought to explore the predictors of these symptoms and to identify other self/parent-reported psychiatric manifestations that occur during the infection. We hypothesized that the prevalence of depressive or anxiety symptoms among pediatric patients with DF would be higher than that among youth controls, and predictors would include pain-related DF symptoms including headache, myalgia, arthralgia, retro-/peri-orbital pain and abdominal pain suggesting a causal link between depressive, anxiety and other psychiatric symptoms and DF infection.*	*Introduction*<br>3
**Methods**			
Study design	4	Present key elements of study design early in the paper<br>*We mentioned "This case-control study involved pediatric patients (cases) admitted at three public tertiary (>100 beds) hospitals in Metro Manila, Philippines, from July to November 2017, during the high transmission of DF cases." in the first sentence of the*	*Study and Sampling Design*<br>3

		study and sampling design. We also added, "Simultaneously, healthy Grade 3 to 12 students, whose age were similar with the cases, 8 to 17 years old, were also recruited to serve as controls," in the same paragraph.	
Setting	5	Describe the setting, locations, and relevant dates, including periods of recruitment, exposure (N/A), follow-up (N/A), and data collection<br>*Patients with DF were admitted in 3 public tertiary (>100 beds) hospitals in Metro Manila, Philippines: San Lazaro Hospital, a referral facility for Infectious/ Communicable Diseases, Quezon City General Hospital and; Pasay City General Hospital while the controls were compared with school-based Grade 3 to Grade 12 students (8 to 17 years old). The collection was done during the rainy season from July to November 2017 in Metro Manila, Philippines.*	*Study and Sampling Design*<br>3
Participants	6	(*a*) *Cohort study*—Give the eligibility criteria, and the sources and methods of selection of participants. Describe methods of follow-up<br>*Case-control study*—Give the eligibility criteria, and the sources and methods of case ascertainment and control selection. Give the rationale for the choice of cases and controls<br>*Paediatric (17 years old and below) who had serology-confirmed or clinically diagnosed DF, who were conscious and able to read and write. Excluded were those who were not able to comply with consent procedures, or with life-threatening comorbidities. Healthy school-based youth controls no current or existing signs and clinical symptoms or diagnosis of DF at the time of interview. Recruitment of the patients and controls was also based on age (age 8–17 years) and grade level (grades 3 to 12) criteria of the Revised Child Anxiety and Depression Scale (RCADS-25), a screening tool for depressive and anxiety symptoms. Eligible participants had no history and/or existing diagnosis of psychiatric and/or medical condition for which they had received medical advice or treatment prior to the interview. Patients with life-threatening comorbidities and controls who were not able to comply with consent procedures were excluded.*<br>*Cross-sectional study*—Give the eligibility criteria, and the sources and methods of selection of participants	*Participant Inclusion and Exclusion Criteria*<br>3
		(*b*) *Cohort study*—For matched studies, give matching criteria and number of exposed and unexposed<br>*Case-control study*—For matched studies, give matching criteria and the number of controls per case<br>*We used the 1:1 ratio (one case patient/ one control) with an assumed odds-ratio of ≥2, power (1-β) of 0.80, 0.05 significance level,* Z_{α}*=1.96. However, we've included more controls.*	*Study and Sampling Design*<br>3
Variables	7	Clearly define all outcomes, exposures (N/A), predictors, potential confounders (N/A), and effect modifiers(N/A). Give diagnostic criteria, if applicable<br>*Outcome or Response variables are: depressive and anxiety symptoms. Explanatory variables or predictors: age, civil status, gender, educational attainment or employment status, family monthly income and family, self DF history, admitting diagnosis, serologic test results (NS1Ag and BLOT: IgG and IgM), platelet count, DF phase (acute: febrile to critical and recovery phase) and clinical symptoms.*	*Explanatory variables and Response variables*<br>5-6

Data sources/ measurement	8*	For each variable of interest, give sources of data and details of methods of assessment (measurement). Describe comparability of assessment methods if there is more than one group<br>*We used consistent pre-determined instructions and questions using structured forms. We used RCADS-25, a valid and standardized assessment toll for screening depressive and anxiety symptoms in children and adolescents, among cases and controls.*	*Ethical considerations and data collection procedures*	3
Bias	9	Describe any efforts to address potential sources of bias<br>*To avoid bias, interviews were done with a consistent pre-determined instructions and questions using structured forms and pre-tested self-report questionnaire. This was done to expect a fairly consistent data from one participant to another.*	*Ethical considerations and data collection procedures*	NA
Study size	10	Explain how the study size was arrived at<br>*All the patients in the three hospitals during the data collection period (July to November) were recruited in the study based from the inclusion criteria (grades 3 to 12 and 8 to 17 years old).*		3
Quantitative variables	11	Explain how quantitative variables were handled in the analyses. If applicable, describe which groupings were chosen and why<br>*Explanatory variables or predictors were divided into three categories: socio-demographic profile, clinical parameters and clinical data. Each of the variable was divided into categories. For linear regression analysis, the categorical variables were transformed to dummy variables [i.e., 0 or 1]to identify the predictors of depressive and anxiety symptoms subscales which are continuous data (t-scores).*	*Statistical and Data Analysis*	5
Statistical methods	12	(*a*) Describe all statistical methods, including those used to control for confounding<br>*Multiple linear regression analysis was done by inputting all explanatory variables in the model using a stepwise method in forward selection to identify significant (P < 0.05) predictors of depressive and anxiety symptoms among patients with DF.*	*Statistical and Data Analysis*	5
		(*b*) Describe any methods used to examine subgroups and interactions<br>*We compared the groups: paediatric patients and youth controls by their t-scores n scores in depressive and anxiety subscales using independent samples t-test. We also conducted chi-square analyses and odds-ratio in the prevalence of pediatrics and controls with and without borderline or clinical depressive and anxiety symptoms*	*Statistical and Data Analysis*	5
		(*c*) Explain how missing data were addressed<br>*Missing responses were addressed during the interview, thus, there's a 100% completion rate among participants as ensured by the primary and co-investigators*	*Statistical and Data Analysis*	5
		(*d*) *Cohort study*—If applicable, explain how loss to follow-up was addressed<br>*Case-control study*—If applicable, explain how matching of cases and controls was addressed<br>*We matched the patients with controls according to the total number of patients but we failed to match them by the frequency in each age category, gender, grade level, etc.*<br>*Cross-sectional study*—If applicable, describe analytical methods taking account of sampling strategy	*Study and Sampling Design*	5

		(*e*) Describe any sensitivity analyses<br>*We did not do any sensitivity analyses in this study. However, we did expert validation and measured the internal consistency of the Filipino version of RCADS child and parent forms using Cronbach's alpha among clinical and school-based samples.*		5

Results

Participants	13*	(a) Report numbers of individuals at each stage of study—eg numbers potentially eligible, examined for eligibility, confirmed eligible, included in the study, completing follow-up, and analysed<br>*Initially, 625 participants (pediatric patients n = 321; youth controls n = 304) were recruited in the study, but only 485 (pediatric patients n = 225; youth controls n = 260) were found eligible, complied to the informed consent procedures and participated in this study.*	*Socio-demographic Profile, clinical parameters and symptoms*	5-6
		(b) Give reasons for non-participation at each stage<br>*We have excluded those who were not eligible and did not comply to the informed consent procedures*	*Socio-demographic Profile, clinical parameters and symptoms*	5-6
		(c) Consider use of a flow diagram		
Descriptive data	14*	(a) Give characteristics of study participants (eg demographic, clinical, social) and information on exposures and potential confounders<br>*Please see Table 1. Socio-demographic profile, clinical parameters and clinical symptoms among paediatric and youth controls*	*Socio-demographic Profile, clinical parameters and symptoms*	5-8
		(b) Indicate number of participants with missing data for each variable of interest<br>*All participants had complete responses. We have the same number of participants in all response or outcome variables.*	*Socio-demographic Profile, clinical parameters and symptoms*	5-8
		(c) *Cohort study*—Summarise follow-up time (eg, average and total amount)		5-8
Outcome data	15*	*Cohort study*—Report numbers of outcome events or summary measures over time		
		Case-control study—Report numbers in each exposure category, or summary measures of exposure<br>*We reported the prevalence and mean scores obtained by the participant in depressive and anxiety subscales. Please see Table 2 Prevalence and mean score difference of depressive and anxiety symptoms between pediatric patients with DF and youth controls*	*Mean score difference difference of depressive and anxiety symptoms between pediatric patients with DF and youth controls*	5-8
		Cross-sectional study—Report numbers of outcome events or summary measures		
Main results	16	(*a*) Give unadjusted estimates and, if applicable, confounder-adjusted estimates and their precision (eg, 95% confidence interval). Make clear which confounders were adjusted for and why they were included		5-8

		We did not include unadjusted estimates and confounder-adjusted estimates with precision in our study.	
		(*b*) Report category boundaries when continuous variables were categorized<br>*We did not categorize the outcome variables.*	5-8
		(*c*) If relevant, consider translating estimates of relative risk into absolute risk for a meaningful time period<br>*We did not estimate relative risk or absolute risk in the study.*	5-8
Other analyses	17	Report other analyses done—eg analyses of subgroups and interactions, and sensitivity analyses<br>*Multiple linear regression analysis found significant regression equations in depressive and anxiety symptoms subscales domains among paediatric patients with DF*<br>*Patients' reported psychiatric manifestations were analyzed using content analysis, a method that can identify patterns across qualitative data (words or phrases) that can be counted (frequency) for quantitative analyses*	*Predictors of depressive and anxiety symptoms Self/parent-reported psychiatric manifestations*<br>5-8
**Discussion**			
Key results	18	Summarise key results with reference to study objectives<br>*The first paragraph of the discussion summarizes the key results*	*Discussion*<br>8-9
Limitations	19	Discuss limitations of the study, taking into account sources of potential bias or imprecision. Discuss both direction and magnitude of any potential bias<br>*We mentioned the limitations in the last paragraph of the discussion. We mentioned failure to match cases with controls, income and hospital setting, time of collection and lack of further psychiatric assessment by a psychiatrist*	*Discussion*<br>8-9
Interpretation	20	Give a cautious overall interpretation of results considering objectives, limitations, multiplicity of analyses, results from similar studies, and other relevant evidence<br>These are our interpretation sin the key results of our study:<br>1. *The prevalence of depressive and anxiety symptoms among pediatric patients with DF was significantly higher than that among youth controls.*<br>2. *The difference between the prevalence of depressive and anxiety symptoms between pediatric patients and youth controls may have brought about by the presence of pain related to myalgia and arthralgia, age, ≤2 days of hospitalization and family history of DF.*<br>3. *The results confirm that similar with adult DF in previous studies, pediatric patients with DF also exhibit psychiatric manifestations like irritable mood/irritability, visual hallucination, agitation and aggressiveness during DF infection*	*Discussion*<br>8-9
Generalisability	21	Discuss the generalisability (external validity) of the study results<br>*Some generalisabilities of the results from this study were discussed especially in the larger context pediatric patients with DF.*	*Discussion*<br>5-8

**Other information**			
Funding	22	Give the source of funding and the role of the funders for the present study and, if applicable, for the original study on which the present article is based<br>*This study was supported by the Japan Society for the Promotion of Science (JSPS) Grant-in-Aid for Scientific Research (16H05750, 17H01624) and JSPS Bilateral Joint Research Projects which had no role in the design, data collection, statistical analysis and writing of this manuscript.*	*Funding*<br>9

*Give information separately for cases and controls in case-control studies and, if applicable, for exposed and unexposed groups in cohort and cross-sectional studies.

Note: An Explanation and Elaboration article discusses each checklist item and gives methodological background and published examples of transparent reporting. The STROBE checklist is best used in conjunction with this article (freely available on the Web sites of PLoS Medicine at http://www.plosmedicine.org/, Annals of Internal Medicine at http://www.annals.org/, and Epidemiology at http://www.epidem.com/). Information on the STROBE Initiative is available at www.strobe-statement.org.

English certificates

We certify that the following article

On the track of new endoscopic alternatives for the treatment of selected gastric GISTs – a pilot study.

Artur Raiter, Katarzyna Monika Pawlak *, Katarzyna Kozłowska-Petriczko, Jan Petriczko, Joanna Szełemej
Anna Wiechowska-Kozłowska

has undergone English language editing by MDPI. The text has been checked for correct use of grammar and common technical terms, and edited to a level suitable for reporting research in a scholarly journal.
MDPI uses experienced, native English speaking editors. Full details of the editing service can be found at https://www.mdpi.com/authors/english.

MDPI Multidisciplinary Digital Publishing Institute

Basel, Switzerland
May 2021

AUTHORservices

TEFLEN TRAINING COLLEGE

INTERNATIONAL ACCREDITATION & RECOGNITION COUNCIL

TEACHING ENGLISH TO SPEAKERS OF OTHER LANGUAGES (TESOL)
TEACHING ENGLISH AS A FOREIGN LANGUAGE (TEFL)

IARC

Acknowledges The Completion Of The TTC, 150 Hour Master TESOL Course

TEFL-TESOL CERTIFICATION IS HEREBY GRANTED TO

Raquel Ortega

On The 1st Day Of December In The Year 2016

PETER JAMES STEELE
PRINCIPAL

TIMOTHY TOWNSEND
COURSE DIRECTOR

DOCEO ANGLICUS TEFL TESOL

T275477
CERTIFICATION VALIDATION

A
AWARDED GRADE
